# Invasive Right Anomalous Coronary Arteries Assessment: Intravascular Ultrasound and Adenosine vs Dobutamine Fractional Flow Reserve

**DOI:** 10.1016/j.jacadv.2025.102526

**Published:** 2026-01-24

**Authors:** Anselm W. Stark, Adriana Ferroni, René L. Mathey-de-l’Endroit, Ryota Kakizaki, Marius R. Bigler, Flavio Giuseppe Biccirè, Marc Ilic, Isaac Shiri, Andreas Haeberlin, Matthias Siepe, Stephan Windecker, Lorenz Räber, Christoph Gräni

**Affiliations:** a Department of Cardiology, Inselspital, Bern University Hospital, University of Bern, Bern, Switzerland; b Department of Cardiac Surgery, Inselspital, Bern University Hospital, University of Bern, Bern, Switzerland

**Keywords:** AAOCA, ACAOS, dobutamine, FFR, IVUS, R-AAOCA

## Abstract

**Background:**

In the right anomalous aortic origin of a coronary artery (R-AAOCA), invasive fractional flow reserve during dobutamine (FFR_Dobutamine_) allows to assess dynamic interarterial/intramural compression that may lead to ischemia. However, FFR_Dobutamine_ requires expertise and is procedurally complex. Therefore, a simpler alternative is needed, but it remains uncertain whether routine intravascular ultrasound (IVUS) and standard FFR during adenosine (FFR_Adenosine_) can reliably determine hemodynamic relevance.

**Objectives:**

This study aimed to compare the diagnostic accuracy of FFR_Adenosine_ and IVUS-based minimal lumen area (IVUS-MLA) against the reference standard of FFR_Dobutamine_ for identifying hemodynamically relevant R-AAOCA.

**Methods:**

Consecutive patients of a single center were prospectively enrolled over 5 years (June 2020-June 2025) with R-AAOCA and interarterial/intramural course. All patients underwent FFR_Dobutamine_, FFR_Adenosine_, and resting IVUS-MLA of the proximal anomalous segment. Hemodynamically relevant R-AAOCA was defined as FFR_Dobutamine_ ≤0.80. Diagnostic performance was assessed using receiver-operating characteristic analysis.

**Results:**

A total of 73 patients (mean age 51 ± 13 years; 34% female) were included. Seventeen patients (23%) had hemodynamically relevant R-AAOCA. FFR_Adenosine_ ≤0.80 predicted FFR_Dobutamine_ ≤0.80 with 100% specificity and positive predictive value, 29% sensitivity and 82% negative predictive value (area under the curve: 0.81). IVUS-MLA ≤5.5 mm^2^ predicted hemodynamic relevance with 100% sensitivity and negative predictive value, 68% specificity, and 49% positive predictive value (area under the curve: 0.88).

**Conclusions:**

In adults with newly diagnosed R-AAOCA and interarterial and intramural course, one-quarter were hemodynamically relevant. FFR_Adenosine_ could reliably rule-in, while IVUS-MLA could effectively rule-out relevant R-AAOCA, potentially reducing the need for FFR_Dobutamine_ testing.

Right anomalous aortic origin of a coronary artery (R-AAOCA) is a rare congenital condition, which is diagnosed more frequently in the current era of increasingly used invasive and noninvasive anatomical imaging. A specific subgroup of R-AAOCA, notably those exhibiting both an interarterial (between the aorta and pulmonary artery) and an intramural (within the aortic wall) course, is linked to an increased risk of ischemia and sudden cardiac death.^1^, ^2^, ^3^ However, identifying those at higher risk for ischemia is challenging,^4^, ^5^, ^6^ and physicians frequently face the dilemma of whether surgical correction is necessary or not based on anatomy alone.^4^^,^^7^, ^8^, ^9^, ^10^ Therefore, to support this decision-making process, the European guidelines^11^ and U.S. guidelines^12^ highlight the role of functional testing to evaluate hemodynamic relevance.^13^^,^^14^ Among all different noninvasive and invasive functional testing, fractional flow reserve during dobutamine (FFR_Dobutamine_) testing represents the gold standard to assess both fixed and dynamic components (ie, stress-induced lumen deformation) in R-AAOCA with an interarterial and intramural course and allows minimizing false negative rates compared to noninvasive tests.^13^, ^14^, ^15^, ^16^, ^17^ However, FFR_Dobutamine_ is resource-intensive and procedurally complex, it requires prolonged protocols, continuous infusions, and additional personnel, and therefore is typically only available in specialized centers, has procedural-related risk and is uncomfortable for the patient. A simpler alternative is needed, and it remains uncertain whether intravascular ultrasound (IVUS) or FFR during adenosine (FFR_Adenosine_) can reliably determine hemodynamic relevance. Moreover, this question is particularly pertinent when R-AAOCA is discovered during standard coronary angiography, where prolonged procedures, including dobutamine testing, might not be feasible within the same session. Therefore, our aim was to assess the predictive value of straightforward invasive FFR_Adenosine_ and/or IVUS for hemodynamic relevance in consecutive adult patients with R-AAOCA and interarterial and intramural course against the reference standard of invasive FFR_Dobutamine_.

## Material and methods

### Study population

Consecutive patients with any newly detected coronary artery anomaly presenting at our specialized coronary artery anomaly clinic between June 2020 and June 2025 were prospectively enrolled in the noninvasive anatomical assessment for ruling out hemodynamically relevant coronary artery anomalies (NARCO) study.^18^ An a priori power analysis indicated that a minimum of 38 patients would be required to achieve sufficient statistical power. However, we deliberately continued enrolling all eligible patients throughout the 5-year study period, ultimately including 73 patients, ensuring a final cohort size that enhanced the statistical reliability. Our specialized coronary anomaly clinic includes individuals with any coronary artery anomaly (eg, symptomatic patients, incidental findings on imaging, or referrals from regional/national/international centers). All patients undergo coronary computed tomography angiography, if not already provided, for a clear classification of the anomaly. Inclusion criteria for this specific study included the presence of an R-AAOCA with an interarterial and intramural course, age ≥18 years, and provision of written informed consent. For this study, all patients underwent invasive coronary angiography using FFR_Adenosine_ and FFR_Dobutamine_ as well as IVUS. Local ethics approval is available (KEK 2020-00841), and participants provided written, informed consent. The study was registered with ClinicalTrials.gov (NCT04475289).

### Intravascular hemodynamic assessment

Coronary angiography was performed from a radial access or femoral access, if initial radial access was not successful. Following intubation with a 6 or 6.5 French guiding catheter, the study protocol included pressure monitoring using a 0.0014-inch pressure monitoring angioplasty guidewire (PressureWire X Guidewire, Abbott), which was calibrated and positioned distal to the intramural segment. FFR_Adenosine_ was assessed under maximal hyperemia with continuous intravenous adenosine infusion for 2 minutes with 140μg/kg/min and calculated as the ratio of mean distal pressure (after the intramural segment) to mean aortic pressure.

For the simulated exercise condition, dobutamine-volume-atropine challenge was performed. Specifically, dobutamine was administered intravenously at a rate of 20 μg/kg/min gradually increased, followed by at least 4 minutes at a rate of 40 μg/kg/min. Following administration of 3 L of saline solution, to counteract reduction of preload caused by dobutamine, 1 mg of atropine was administered on top of dobutamine to achieve maximal heart rate (min. 85% of predicted maximal heart rate or above). Stationary FFR_Dobutamine_ was then measured distal to the intramural segment. Following the acquisition of FFR_Dobutamine_ results, the pressure wire was pulled back, and the results were adjusted for potential drifts. Subsequently, the dobutamine infusion was stopped, and esmolol (40 mg bolus injections) was given to reduce the heart rate to normal levels. Hemodynamic relevance was defined as FFR_Dobutamine_ ≤0.8.

### Intravascular ultrasound assessment

IVUS was conducted at rest, before adenosine and dobutamine administration, in a standard manner with an automated pullback (1 mm/s) using a 40 MHz rotational transducer (Boston Scientific), with images being captured at a rate of 30 frames per second. The IVUS pullback was initiated by positioning the probe distally to the intramural segment (ie, fully circular lumen) and retracting the probe until only the aorta was visible. The intramural course was identified as the region spanning between the ostium and the first round lumen distal to the ostium. The minimal lumen area (IVUS-MLA) was identified within the intramural course. The maximal lumen narrowing (IVUS-MLN) was defined as: 1 − (IVUS-MLA/distal reference) × 100, with the largest visible distal area defined as the distal reference. The elliptic ratio was defined as the ratio of the major axis to the minor axis of the IVUS-MLA. All analyses were performed with the AIVUS-CAA software.^19^

### Follow-up and outcome definition

Follow-up assessments were performed through yearly clinical evaluations, telephone interviews, and clinical reports. Major cardiac adverse events were defined as sudden cardiac death/survived sudden cardiac death, sustained ventricular tachycardia lasting more than 30 seconds, myocardial infarction, revascularization of the anomalous vessel, or implantation of an implantable cardioverter-defibrillator.

### Statistical analysis

The normality of continuous data was assessed using a Shapiro-Wilk test and visual inspection of Q-Q plots. Normally distributed continuous variables were presented as mean ± SD, while non-normally distributed variables were reported as median and 25th to 75th percentile. Categorical variables were expressed as counts and percentages. Variables were compared using the Student’s *t*-test for normally distributed data and the Wilcoxon signed rank test for non-normally distributed data. Categorical variables were compared using chi-square tests, with Fisher exact test used when expected cell counts were <5. *P* values were adjusted for multiple testing using a false discovery rate test by Benjamini & Hochberg procedure.

The reference standard for hemodynamic relevant R-AAOCA was defined as FFR_Dobutamine_ ≤0.80. The cutoff for FFR_Adenosine_ was as well ≤0.80. Simple logistic regression analysis was conducted with hemodynamic relevance (FFR_Dobutamine_ ≤0.8) as the binary response variable and continuous IVUS anatomical features and FFR_Adenosine_ as explanatory variables.

Significant predictors from logistic regression models were further evaluated using receiver-operating characteristics (ROC) and area under the curve (AUC). For each ROC model, all possible cutoff values were evaluated through iterative selection by maximizing sensitivities and specificities with Youden’s method, maximizing sensitivity alone and further maximizing specificity alone. Incorporating the derived cutoff values into predictive models, performance metrics including sensitivity, specificity, positive predictive value (PPV), negative predictive value (NPV), and accuracy were comprehensively evaluated to assess the effectiveness of the selected cutoff values in discriminating between positive and negative cases within the study population.

To assess the robustness and generalizability of these cutoff values, an internal validation was performed using repeated random split-sample testing. The data set was repeatedly and randomly split without replacement into a training set (79%, n = 58) and a test set (21%, n = 15) using stratified sampling to preserve the ratio of hemodynamically relevant cases. For each of 100 iterations (each with a unique random seed), ROC analysis on the training set determined the 3 cutoffs (Youden’s, max sensitivity, max specificity), which were then applied to the held-out test set to calculate performance metrics. The results across all 100 iterations were averaged to provide robust estimates of model performance (Supplemental Table 2). All statistical analyses were conducted using R (version 4.2.3) and the pROC and caret libraries.

## Results

### Demographics

**Figure 1:**
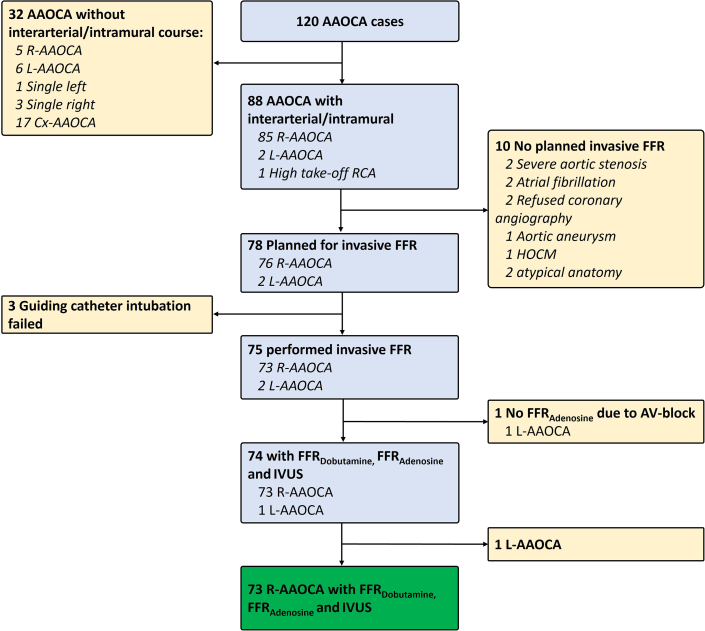
**Flowchart** AAOCA = anomalous aortic origin of a coronary artery; Cx-AAOCA = circumflex anomalous aortic origin of a coronary artery; FFR = fractional flow reserve; FFR_Adenosine_ = fractional flow reserve during adenosine; FFR_Dobutamine_ = fractional flow reserve during dobutamine-atropine-volume challenge; HOCM = hypertrophic obstructive cardiomyopathy; IVUS = intravascular ultrasound during resting conditions; L-AAOCA = left anomalous aortic origin of a coronary artery; R-AAOCA = right anomalous aortic origin of a coronary artery; RCA = right coronary artery.

Of the total 120 patients screened for AAOCA, 78 showed an interarterial and intramural course according to computed tomography and were scheduled for invasive coronary angiography. Of these, intubation of the anomalous vessel with a guiding catheter failed in 3 patients. Testing had to be aborted in one patient due to a transient atrioventricular block during adenosine administration, and one patient with L-AAOCA was excluded. Finally, a total of 73 consecutive patients with newly diagnosed with R-AAOCA and an interarterial and intramural course were included in this study and analyzed (Figure 1). The average age was 51 ± 13 years, with 34% women. No significant differences were observed in the baseline characteristics between the hemodynamic relevant and nonrelevant groups (Table 1).

**Table 1:** Baseline Characteristics and Diagnostic Parameters Between Hemodynamically Relevant and Nonrelevant Cases

	All (N = 73)	Hemodynamic Relevant (FFR_Dobutamine_ ≤0.8) (n = 17)	Hemodynamic Nonrelevant (FFR_Dobutamine_ >0.8) (n = 56)	*P* Value (Adjusted)
Age, y	51 ± 13	49 ± 13	53 ± 13	0.54
Female	25 (34%)	8 (47%)	17 (30%)	0.54
BMI, kg/m^2^	25.3 (22.8-29.3)	24.9 (21.3-27.2)	26.1 (23.6-30.5)	0.22
History of dyslipidemia	34 (47%)	7 (41%)	27 (48%)	1.00
History of diabetes mellitus	3 (4%)	0 (0%)	3 (5%)	NA
History of tobacco	31 (42%)	9 (53%)	22 (39%)	0.65
History of hypertension	25 (34%)	5 (29%)	20 (36%)	0.93
Family history of CAD	22 (30%)	5 (29%)	17 (30%)	1.00
Family history of SCD	5 (7%)	0 (0%)	5 (9%)	NA
Asymptomatic	13 (18%)	3 (18%)	10 (18%)	1.00
Typical angina pectoris	38 (56%)	9 (53%)	29 (52%)	1.00
Atypical angina pectoris	28 (38%)	5 (29%)	23 (41%)	077
Dyspnea	19 (26%)	6 (35%)	13 (23%)	0.71
Syncope	5 (7%)	3 (18%)	2 (4%)	0.26
Syncope during exercise	2 (3%)	2 (12%)	0 (0%)	NA
CAD	13 (19%)	2 (12%)	11 (20%)	1.00
CAD ≥50% stenosis in anomalous vessel	4 (5%)	1 (6%)	3 (5%)	1.00
Dominance				0.31
Right	69 (95%)	15 (88%)	54 (96%)	
Balanced	1 (1%)	0 (0%)	1 (2%)	
Left	3 (4%)	2 (12%)	1 (2%)	
Access site				1.00
Radial	70 (96%)	16 (94%)	54 (96%)	
Femoral	3 (4%)	1 (6%)	2 (4%)	
IVUS-MLA (mm^2^)	5.6 (4.5-7.0)	4.1 (3.3-5.0)	6.0 (4.8-7.7)	**<0.001**
IVUS elliptic ratio	3.3 (2.5-3.9)	4.1 (3.3-5.4)	3.6 (2.4-3.6)	**0.008**
IVUS-MLN (%)	57 (46-63)	62 (59-73)	54 (45-60)	**0.003**
IVUS-MLN ≥50% (n)	51 (70%)	15 (88%)	36 (64%)	0.22
FFR_Adenosine_	0.92 (0.89-0.95)	0.84 (0.80-0.90)	0.93 (0.91-0.95)	**<0.001**
FFR_Dobutamine_	0.87 (0.81-0.91)	0.76 (0.68-0.80)	0.89 (0.87-0.92)	**<0.001**
Heart rate during stress (beats/min)	153 (145-160)	150 (147-157)	154 (144-161)	0.76
Mean aortic pressure during stress (mm Hg)	96 ± 21	99 ± 25	95 ± 20	0.76
Underwent stenting	2 (3%)	2 (12%)	0 (0%)	NA
Underwent surgery	10 (14%)	10 (59%)	0 (0%)	NA
Sports restriction	8 (11%)	3 (18%)	5 (9%)	0.76
Start aspirin	20 (27%)	12 (71%)	8 (14%)	**<0.001**
Start beta-blocker	8 (11%)	5 (29%)	3 (5%)	**0.044**
Start statin	13 (18%)	4 (24%)	9 (16%)	0.85

Comparisons within this table were adjusted using the Benjamini & Hochberg false discovery rate (FDR) procedure.

BMI = body mass index; CAD = coronary artery disease; FFR_Adenosine_ = fractional flow reserve during adenosine; FFR_Dobutamine_ = fractional flow reserve during dobutamine-atropine-volume challenge; IVUS = intravascular ultrasound; IVUS-MLA = minimal lumen area during rest; IVUS-MLN = maximal lumen narrowing during rest; SCD = sudden cardiac death.

### Hemodynamic changes during dobutamine stress

There were no significant differences in change of heart rate, systolic, mean, or diastolic aortic pressure between the hemodynamically relevant and nonrelevant groups (Supplemental Table 1). Notably, heart rate increased from a median of 74 beats/min (IQR: 67-79 beats/min) to a median of 150 beats/min (IQR: 147-157 beats/min) in the hemodynamic relevant group, and from a median of 76 (IQR: 69-89) beats/min to a median of 151 (IQR: 144-161) beats/min in the nonrelevant group. In the hemodynamic relevant group, 11/17 (65%) of patients reached their target heart rate of 85% of predicted maximum and in the hemodynamic nonrelevant group 44/56 (79%) (*P* = 0.40). Aortic pressure increased from 109 ± 22 mm Hg to 147 ± 30 mm Hg in the relevant group, and from 113 ± 21 mm Hg to 143 ± 25 mm Hg in the nonrelevant group (*P* = 0.28).

The hemodynamically relevant group exhibited a greater reduction in FFR, with median FFR_Adenosine_ decreasing from 0.84 (IQR: 0.80-0.90) to median FFR_Dobutamine_ 0.76 (IQR: 0.68-0.80), compared to the nonrelevant group, where FFR_Adenosine_ decreased from 0.93 ± 0.04 to FFR_Dobutamine_ 0.89 ± 0.04 (*P* = 0.003).

Average procedure time was 14 minutes for FFR_Adenosine_ and IVUS, and an additional 22 minutes to perform FFR_Dobutamine_.

### FFR_Adenosine_ vs FFR_Dobutamine_

**Figure 3:**
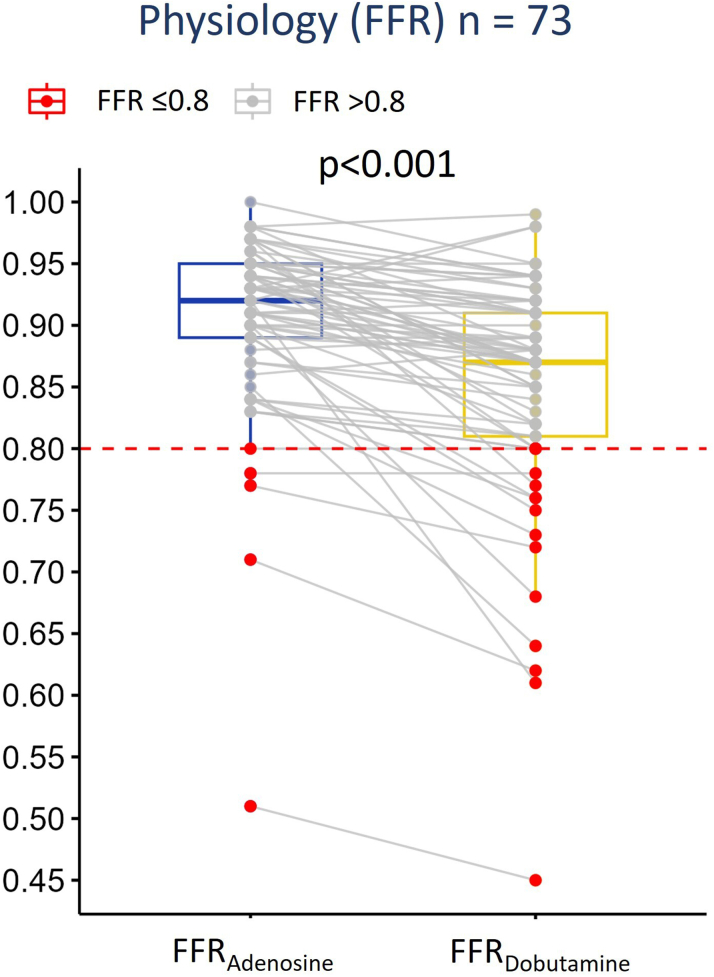
**Dynamic Changes During Dobutamine-Atropine-Volume Challenge** Hemodynamic relevant patients as assessed by any FFR ≤0.8 are indicated in red. All patients with an FFR_Adenosine_ ≤0.8 remained ≤0.8 during FFR_Dobutamine_. Abbreviations as in Figure 1.

**Figure 4:**
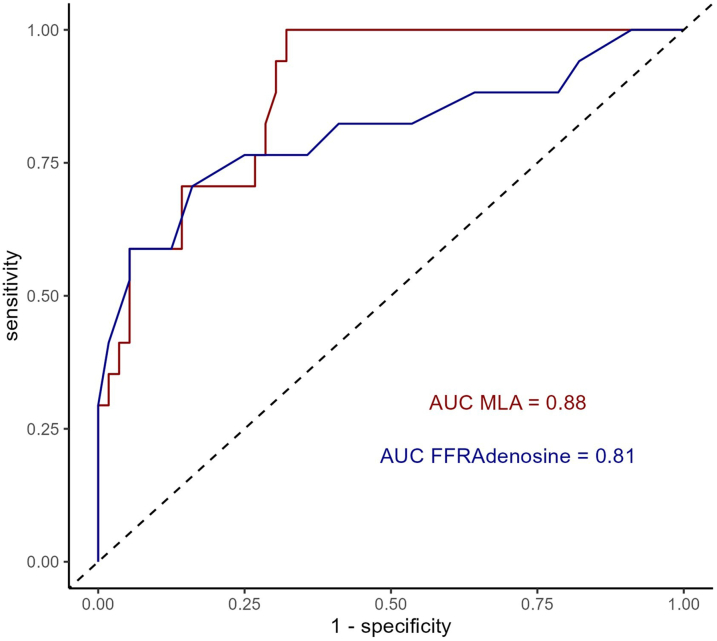
**ROC Analysis of IVUS-MLA and FFR_Adenosine_ Measurements to Predict FFR_Dobutamine_ >0.8** AUC = area under the curve; ROC = receiver-operating characteristics; other abbreviations as in Figures 1 and 2.

Median FFR_Adenosine_ was 0.92 (IQR:_0.89-0.95) and median FFR_Dobutamine_ was 0.87 (IQR: 0.81-0.91) (*P* < 0.001). Five (7%) patients had an FFR_Adenosine_≤0.8 and 17 (23%) patients an FFR_Dobutamine_ ≤0.8. No patients with FFR_Adenosine_ ≤0.8 had normal FFR_Dobutamine_ (Figure 3, Supplemental Table 1). ROC analysis resulted in an AUC of 0.81 and 100% both specificity and PPV, and a sensitivity of 29% and NPV of 82% (Central Illustration, Table 2, Figure 4).

**Central Illustration:**
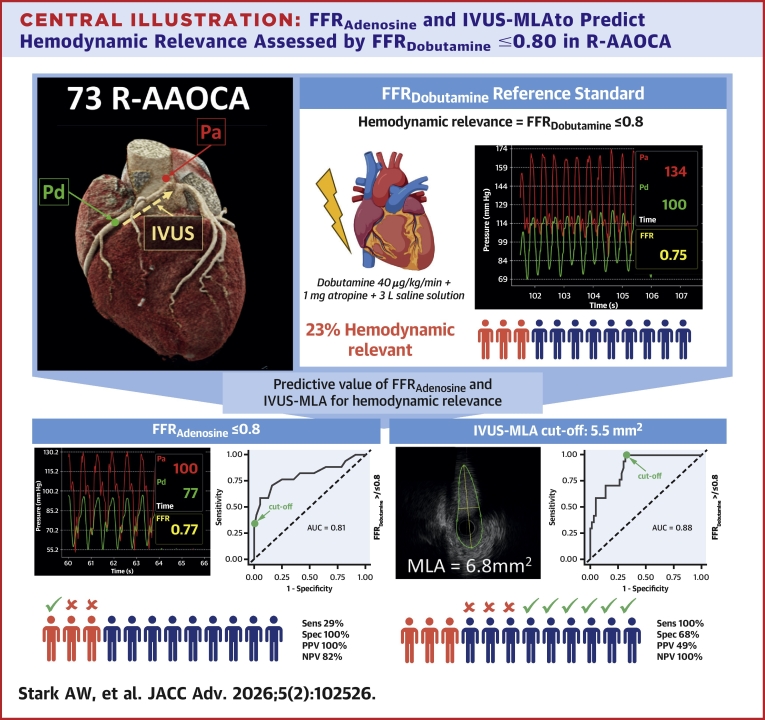
**FFR_Adenosine_ and IVUS-MLA to Predict Hemodynamic Relevance Assessed by FFR_Dobutamine_ ≤0.80 in R-AAOCA** Top panels show IVUS probe positioning with proximal (Pa) and distal (Pd) pressures and define FFR_Dobutamine_ ≤0.80 (23% of patients). Bottom left: FFR_Adenosine_ ≤0.80 had 100% specificity, 100% PPV, 29% sensitivity (AUC: 0.81), useful to rule in hemodynamic relevance. Bottom right: IVUS-MLA cutoff 5.5 mm^2^ had 100% sensitivity, 100% NPV, 68% specificity (AUC: 0.88), useful to rule out hemodynamic relevance. Conclusion: FFR_Adenosine_ confirms, while IVUS-MLA excludes, dobutamine-induced ischemia, potentially reducing need for stress testing. NPV = negative predictive value; PPV = positive predictive value; other abbreviations as in Figures 1, 2, and 4.

**Table 2:** IVUS and FFR_Adenosine_ as Predictors for FFR_Dobutamine_>/≤0.8 With Differing Cutoffs and Their Performance

Predictor	Cutoff Value	AUC	Accuracy (%)	Sensitivity (%)	Specificity (%)	NPV (%)	PPV (%)	True Negative (n)	False Negative (n)	False Positive (n)	True Positive (n)
IVUS-MLA (mm^2^)	5.5	0.88	75	100	68	100	49	38	0	18	17
	5.0		74	76	73	91	46	41	4	15	13
	4.3		86	59	95	88	77	53	7	3	10
	3.4		84	29	100	82	100	56	12	0	5
Elliptic ratio	1.6	0.74	26	100	4	100	24	2	0	54	17
	3.3		62	76	57	89	35	32	4	24	13
	4.2		79	47	89	85	57	50	9	6	8
	5.9		81	18	100	80	100	56	14	0	3
IVUS-MLN (%)	41	0.77	41	100	23	100	28	13	0	43	17
	59		70	76	68	90	42	38	4	18	13
	66		78	47	88	84	53	49	9	7	8
	73		82	24	100	81	100	56	13	0	4
FFR_Adenosine_	0.97	0.81	30	100	9	100	25	5	0	51	17
	0.85		86	59	95	88	77	53	7	3	10
	0.82		84	29	100	82	100	56	12	0	5
	0.80		84	29	100	82	100	56	12	0	5

AUC = area under the curve; NPV = negative predictive value; PPV = positive predictive value; other abbreviations as in Table 1.

### IVUS derived anatomical features to predict hemodynamic relevance

**Figure 2:**
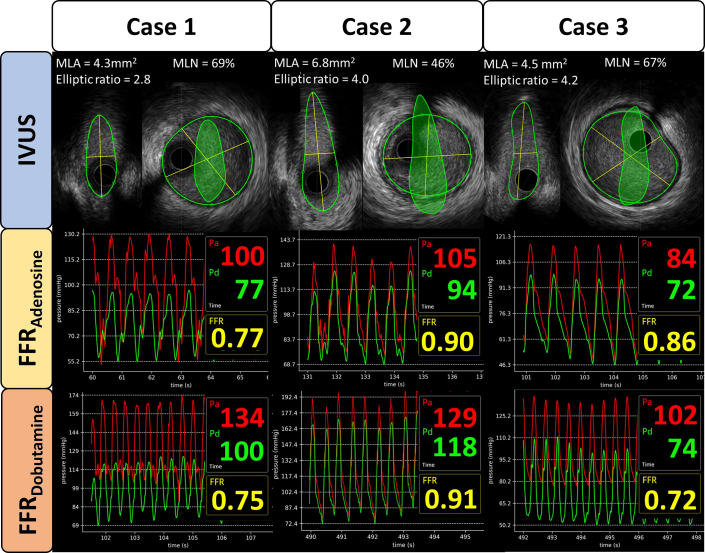
**Patient Examples** Column 1 shows a true positive case predicted by FFR_Adenosine._ The patient presents with an IVUS-MLA ≤5.5 mm^2^ and an FFR_Adenosine_ ≤0.8. The FFR_Dobutamine_ ≤0.8 showed a hemodynamic relevant R-AAOCA. Column 2 shows a true negative case predicted by IVUS-MLA. The individual presents with an IVUS-MLA >5.5 mm^2^ and additionally FFR_Adenosine_ >0.8. FFR_Dobutamine_ demonstrates a hemodynamic nonrelevant vessel with FFR >0.8. Column 3: A case where FFR_Dobutamine_ is necessary for the decision. The patient presents with an IVUS-MLA <5.5 mm^2^ and an FFR_Adenosine_ >0.8. The FFR_Dobutamine_ demonstrates a hemodynamic vessel with FFR ≤0.8. MLA = minimal lumen area; MLN = maximal lumen narrowing; Pa = arterial pressure; Pd = distal pressure; other abbreviations as in Figure 1.

The IVUS-MLA was significantly smaller in the hemodynamic relevant group (4.1 mm^2^ vs 6.0 mm^2^; *P* < 0.001). Similarly, the IVUS-MLN was higher in the hemodynamic relevant group (62% vs 54%; *P* = 0.003), while the elliptic ratio was also greater in the hemodynamic relevant group (4.1 vs 3.6; *P* = 0.008). Logistic regression analysis showed that IVUS-MLA (OR: 0.24; IQR: 0.09-0.47; *P* < 0.001), elliptic ratio (OR: 2.23; IQR: 1.38-3.91; *P* = 0.002), and IVUS-MLN (OR: 1.1; IQR: 1.04-1.18; *P* = 0.004) could predict hemodynamic relevance (Supplemental Figure 1). For ROC analysis, cutoffs of the variables were chosen to maximize sensitivity. A cutoff of 5.5 mm^2^ for IVUS-MLA (AUC: 0.88) resulted in 100% both sensitivity and NPV, 68% specificity and 49% PPV (below cutoff higher chance for hemodynamic relevance, above cutoff lower chance), which allowed to identify 38 true negative cases (Central Illustration). For IVUS-MLN (AUC: 0.75), the cutoff of 41% resulted in 100% both sensitivity and NPV, specificity of 23% and PPV of 28%, which lead to a true negative number of 13 patients. Differing cutoffs and the corresponding predictive performance are presented in Table 2, the ROC curves for all IVUS measurements can be found in Figure 4 and Supplemental Figure 2. Additional analysis with 58 patients in the training set and 15 patients in the test set over 100 random seeds, resulted in an AUC of 0.90 ± 0.08 for IVUS-MLA, where a cutoff of 5.0 ± 0.6 mm^2^ had a sensitivity of 99% ± 5% and a NPV of 100% ± 1% and allowed to identify 9 ± 2 true negative with 0 ± 0 false negative cases (Supplemental Table 2). Multidimensional plots for the relationship of FFR_Dobutamine_ with IVUS-MLA, IVUS-MLN, and elliptic ratio can be found in Supplemental Figures 3 to 5. A patient example to visualize the application of these results can be found in Figure 2.

### Treatment

Interdisciplinary decision (ie, interventional cardiologists, cardiovascular imagers, and cardiac surgeons) for revascularization vs conservative treatment was performed in our specialized AAOCA clinic based on testing findings, symptoms of the patient, individual risk (eg, competitive sports activity), and involving the patient. Revascularization was performed in 12 patients (16%): 10 (14%) underwent surgical unroofing, and 2 (3%) received stent implantation (all with FFR_Dobutamine_ ≤0.8). Among the conservatively managed group (n = 4), the median FFR_Dobutamine_ was 0.78 (range: 0.76-0.80), compared to 0.75 (range: 0.45-0.80) in the treatment group (*P* = 0.36). One case of ostial subocclusion, likely due to dissection occurring immediately after the FFR_Dobutamine_ examination, was successfully treated with stent placement.

### Follow-up and outcome

No R-AAOCA associated adverse events occurred at a median follow-up of 2.0 (1.5-3.5) years in the entire cohort.

## Discussion

This study is the largest prospective cohort investigation of R-AAOCA cases with an interarterial and intramural course that were systematically evaluated using FFR_Adenosine_, IVUS vs FFR_Dobutamine_ as the reference standard. Our findings can be summarized as follows: 1) in adult patients with newly diagnosed R-AAOCA, one-quarter of the cases were hemodynamic relevant according to the reference standard FFR_Dobutamine,_ while FFR_Adenosine_ underdiagnosed ischemia in a significant proportion of cases; 2) FFR_Adenosine_ reliably identified patients with hemodynamic relevance due to its high specificity and PPV, while 3) IVUS-MLA effectively ruled-out relevant R-AAOCA with high sensitivity and NPV.

By combining these approaches, most R-AAOCA cases can be evaluated with standard FFR_Adenosine_ or IVUS evaluation as FFR_Adenosine_ effectively identified one-third of all hemodynamically relevant patients, while IVUS-MLA ruled-out two-thirds of the hemodynamically nonrelevant cases. Consequently, FFR_Dobutamine_ is only necessary for a subset of R-AAOCA patients—specifically, those with normal FFR_Adenosine_ results or an IVUS-MLA <5.5 mm^2^. This approach could reduce the need for FFR_Dobutamine_ procedures, which are complex, are associated with a low but non-negligible risk, can only be performed in specialized centers, and can be uncomfortable for patients. The short- to mid-term outcomes were favorable in applying this strategy where only hemodynamic clear relevant patients were treated.

### FFR_Adenosine_ vs FFR_Dobutamine_

While FFR_Adenosine_ is well-established for assessing coronary artery disease (CAD) and accurately reflects “fixed stenosis” in atherosclerotic plaques, it is less effective at detecting lateral compression, which FFR_Dobutamine_ can capture more effectively. This distinction was clearly shown by the differences in FFR results between the 2 agents and the high sensitivity and low specificity of adenosine testing in this clinical setting. Our study aligns with previous research, where similarly significantly lower FFR_Dobutamine_ values compared to FFR_Adenosine_ were reported.^14^^,^^15^^,^^20^ Some differences in hemodynamic relevance across studies may partly arise from variations in patient selection and stress protocols. For example, to counteract pressure drops during dobutamine stress testing, we used a larger saline volume, which was well tolerated without any adverse events.^21^ Other, less studied protocols have used adrenaline as a stress agent, showing mixed results with some cases observing increased FFR under adrenaline.^22^ However, experience with adrenaline as a stress agent is limited, and it is unclear if adrenaline accurately reflects the stress of physical exercise. Of note, only FFR was investigated in this study, and whether iFR might serve as a better parameter to overcome potential overshooting effects (as observed in myocardial bridging) should be evaluated in future studies.^23^

The main advantage of FFR_Adenosine_ is its shorter procedure time, as the administration of saline solution required to achieve sufficiently high blood pressure during FFR_Dobutamine_ is particularly time-consuming and target heart rate or blood pressure may not be achieved. Additionally, the ostial occlusion we observed appeared after the FFR_Dobutamine_ procedure, highlighting a potentially increased risk associated with this method. Therefore, FFR_Adenosine_ may provide a more straightforward and rapid approach for identifying hemodynamically relevant cases at an early stage of the diagnostic examination.

### IVUS findings

IVUS accurately identified patients at low risk for hemodynamic relevance. Using an MLA cutoff of 5.5 mm^2^, IVUS was able to rule-out half of the patients undergoing invasive stress testing due to high-risk anatomy (eg, interarterial and intramural courses). This finding was validated through random seed testing, reinforcing its reliability. This aligns with previous research showing a similar correlation,^14^ and matches data in CAD, where IVUS-MLA proved to be a stronger predictor of FFR than stenosis grade, indicating a high negative but low positive predictive value for IVUS-MLA.^24^^,^^25^ While several other studies have performed IVUS in R-AAOCA,^14^^,^^21^^,^^22^^,^^26^ most of them did not assess the correlation of IVUS findings to FFR_Dobutamine_. IVUS was primarily utilized to illustrate the “pulsatile lumen deformation” of the lumen area during systole compared to diastole in resting conditions, which is of some interest from a pathophysiological perspective yet not clinically relevant.^27^^,^^28^ However, since lumen compression and subsequent ischemia occur primarily during exercise—when aortic pressure rises and heart rate increases—the specific differences in “stress-induced lumen deformation” (ie, the deformation of the lumen from rest to stress) should be further investigated in future research to better understand the underlying mechanisms of R-AAOCA.

### Impact on clinical guidelines

The absence of specific guidelines for R-AAOCA leads to variable clinical practices and inconsistent patient care, as highlighted by a recent European multicenter study.^29^ This is particularly relevant given the growing number of R-AAOCA diagnoses in adults, driven by increased use of invasive angiography and cardiac imaging. Although invasive FFR_Dobutamine_ is the functional gold standard for assessing AAOCA, it remains underrepresented in current guidelines. In our view, its formal inclusion is warranted. However, due to its technical complexity, need for specialized handling, and associated procedural risks, FFR_Dobutamine_ should be reserved for tertiary centers with appropriate expertise and frequent R-AAOCA referrals.

Importantly, as we have shown for the first time, FFR_Adenosine_ and IVUS could reliably and safely defer invasive stress testing in a significant proportion of nonflow-limiting R-AAOCA cases. This makes them especially useful in general cardiology settings when R-AAOCA is incidentally detected during standard coronary angiography. Only a minority of ambiguous or high-risk cases would require referral to specialized centers.

Once this stepwise approach is validated in larger cohorts and linked to clinical outcomes, its incorporation into guidelines will help standardize care, reduce intercenter variability, and enhance diagnostic accuracy and risk stratification in R-AAOCA.

### Study Limitations

Our study has several limitations. It was a single-center study focused exclusively on R-AAOCA, so its findings may not be applicable to the rarer L-AAOCA cases. Additionally, as a tertiary center, there is a potential for selection bias toward more relevant cases. The small number of patients, despite the relatively high case count for this rare disease, limits the reliability of internal validation, and external validation is currently lacking. Only mid-term follow-up data were available. Additionally, we used the same FFR cutoff as for CAD to define hemodynamic relevance, though it is unclear if dynamic stenosis in R-AAOCA can be assessed by the same criteria as fixed stenosis in CAD. It is also important to note that coronary pressure indices do not directly measure myocardial ischemia, which is the true concern. Other factors, like stenosis diameter, length, microvascular dysfunction, collateral circulation, and other morphological features, may also influence outcomes.^30^, ^31^, ^32^ As a limitation, we acknowledge that performing both FFR_Adenosine_ and IVUS may appear complex and time-consuming; however, both are increasingly used in routine invasive coronary assessment, provide complementary information, and remain easier to perform than FFR_Dobutamine_, which is uncomfortable for the patient and more time-consuming and can often not be performed when AAOCA is newly detected. Moreover, the analysis of IVUS images necessitates a certain level of expertise, as the IVUS-MLA may vary in location within the vessel. The development and application of an automatic segmentation tool could standardize and simplify this process, potentially enhancing the accuracy and consistency of assessments as demonstrated by, for example, AVIUS-CAA. Although FFR_Dobutamine_ is considered the gold standard in this clinical setting, it is rather a surrogate, whereas physical exercise would represent the true gold standard. As the latter is not routinely feasible during catheterization, future approaches should aim to better refine the assessment of the complex pathophysiology of ischemia in R-AAOCA—for example, by comparing different stress protocols incorporating adrenaline, handgrip, or other stimuli. A more advanced and technical alternative could involve the use of digital or physical twins^33^ based on invasive data to simulate exercise conditions beyond those that can be directly measured during catheterization.

## Conclusions

In adults with newly diagnosed R-AAOCA and an interarterial and intramural course, one-quarter of the cases were hemodynamically relevant. While FFR_Adenosine_ testing ruled-in hemodynamically relevant R-AAOCA with high positive predictive value, IVUS was able to rule-out relevant R-AAOCA with high NPV. Therefore, after further validation, FFR_Dobutamine_ assessment may be considered to cases limited by an MLA below cutoff and a negative FFR_Adenosine_.

Perspectives **COMPETENCY IN MEDICAL KNOWLEDGE:** In adults with R-AAOCA with interarterial and intramural course one-quarter was hemodynamically relevant, FFR_Adenosine_ and IVUS-MLA offered reliable simple options for ruling-in and ruling-out a high proportion of patients with regard to hemodynamic relevance and potentially reducing the need for FFR_Dobutamine_ to cases with an MLA below cutoff and negative FFR_Adenosine_.

**TRANSLATIONAL OUTLOOK:** Future studies should investigate long-term clinical outcomes using FFR_Adenosine_ and IVUS-guided management strategies in larger, multicenter cohorts to confirm safety and efficacy.

## Funding support and author disclosures

Dr Gräni has received funding from the Swiss National Science Foundation, 10.13039/501100013348InnoSuisse, Center for Artificial Intelligence in Medicine University Bern, GAMBIT foundation, 10.13039/100008273Novartis Foundation for Medical-Biological Research, Swiss Heart Foundation, outside of the submitted work; and has served as Editor-in-Chief of *The International Journal of Cardiovascular Imaging*, Springer. Dr Häeberlin has received travel fees/educational grants from 10.13039/100004374Medtronic, 10.13039/501100005035Biotronik, 10.13039/100000046Abbott, and 10.13039/100004320Philips/Spectranetics without impact on his personal remuneration; has served as a proctor for Medtronic; has received research grants from the Swiss National Science Foundation, the Swiss Innovation agency 10.13039/501100013348Innosuisse, the Swiss Heart Foundation, the University of Bern, the University Hospital Bern, the Velux Foundation, the Hasler Foundation, the Swiss Heart Rhythm Foundation, and the 10.13039/100008273Novartis Research Foundation; and is a co-founder and CEO of Act-Inno AG. Dr Räber has received funding to the institution from 10.13039/100000046Abbott, 10.13039/100008497Boston Scientific, 10.13039/501100005035Biotronik, 10.13039/100018503Infraredx, 10.13039/100004339Sanofi, and 10.13039/100009857Regeneron and consultation/speaker fees by Abbott, Amgen, Boston Scientific, Biotronik, Gentuity, Novo Nordisk, Medtronic, and Occlutech. Dr Kakizaki has received consulting fee from Infraredx USA, speaker fee from Abbott Medical Japan, Boston Scientific Japan, Philips Japan, Orbusneich Medical, and manuscript writing fee from Orbusneich Medical and Philips Japan, outside the submitted work. Dr Bigler has received a grant from the Bangerter-Rhyner-Foundation. Dr Windecker has received research, travel, or educational grants to the institution without personal remuneration from 10.13039/100000046Abbott, 10.13039/100020297Abiomed, 10.13039/100002429Amgen, 10.13039/100004325AstraZeneca, 10.13039/100004326Bayer, Braun, 10.13039/501100005035Biotronik, Boehringer Ingelheim, Boston Scientific, 10.13039/100001009Bristol Myers Squibb, 10.13039/100018599Cardinal Health, CardioValve, Cordis Medical, Corflow Therapeutics, 10.13039/100008322CSL Behring, 10.13039/501100002336Daiichi Sankyo, 10.13039/100006520Edwards Lifesciences, Farapulse Inc, Fumedica, 10.13039/100020333Guerbet, 10.13039/501100016198Idorsia, 10.13039/100022880Inari Medical, 10.13039/100018503InfraRedx, Janssen-Cilag, Johnson & Johnson, 10.13039/501100023518Medalliance, 10.13039/100008951Medicure, 10.13039/100004374Medtronic, Merck Sharp & Dohm, Miracor Medical, 10.13039/100004336Novartis, 10.13039/501100004191Novo Nordisk, Organon, OrPha Suisse, Pharming Tech., 10.13039/100004319Pfizer, Polares, 10.13039/100009857Regeneron, Sanofi-Aventis, 10.13039/501100011725Servier, Sinomed, 10.13039/501100008645Terumo, 10.13039/501100006484Vifor, and V-Wave; has served as an advisory board member and/or member of the steering/executive group of trials funded by 10.13039/100000046Abbott, 10.13039/100020297Abiomed, 10.13039/100002429Amgen, 10.13039/100004325AstraZeneca, 10.13039/100004326Bayer, Boston Scientific, 10.13039/501100005035Biotronik, 10.13039/100001009Bristol Myers Squibb, 10.13039/100006520Edwards Lifesciences, 10.13039/501100023518MedAlliance, 10.13039/100004374Medtronic, 10.13039/100004336Novartis, Polares, Recardio, Sinomed, 10.13039/501100008645Terumo, and V-Wave with payments to the institution but no personal payments; and is also a member of the steering/executive committee group of several investigator-initiated trials that receive funding by industry without impact on his personal remuneration. All other authors have reported that they have no relationships relevant to the contents of this paper to disclose. Swiss National Science Foundation Grant Number 200871 Noninvasive anatomical assessment for ruling out hemodynamically relevant coronary artery anomalies—A comparison of coronary-CT to invasive coronary angiography (NARCO) to Dr Gräni.
