# Supplementary material for: Invasive Right Anomalous Coronary Arteries Assessment: Intravascular Ultrasound and Adenosine vs Dobutamine Fractional Flow Reserve
Source: JACC Adv. 2026 Jan 24;5(2):102526. doi: 10.1016/j.jacadv.2025.102526 (PMC12860356; doi:10.1016/j.jacadv.2025.102526)
Supplement: Supplemental Tables 1 and 2, Supplemental Figures 1 to 5 [file mmc1.docx]

**Supplemental Figures**

**Supplemental Figure 1:** Logistic regression for different IVUS variables and FFR_Adenosine_. Abbreviations: FFR = Fractional flow reserve, FFR_Dobutamine_ = Fractional flow reserve during dobutamine-atropine-volume challenge


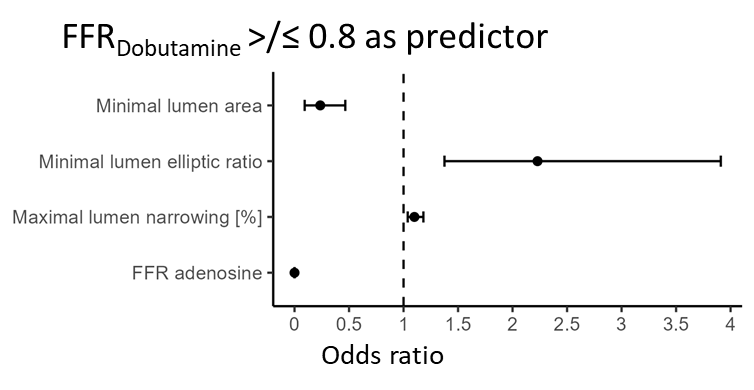


**Supplemental Figure 2:** ROC analysis of different anatomical IVUS measurements during rest to predict patients with an FFR_Dobutamine_ >0.8. Abbreviations: AUC = Area under the curve, IVUS = intravascular ultrasound during rest, MLN = Maximal lumen narrowing during rest.


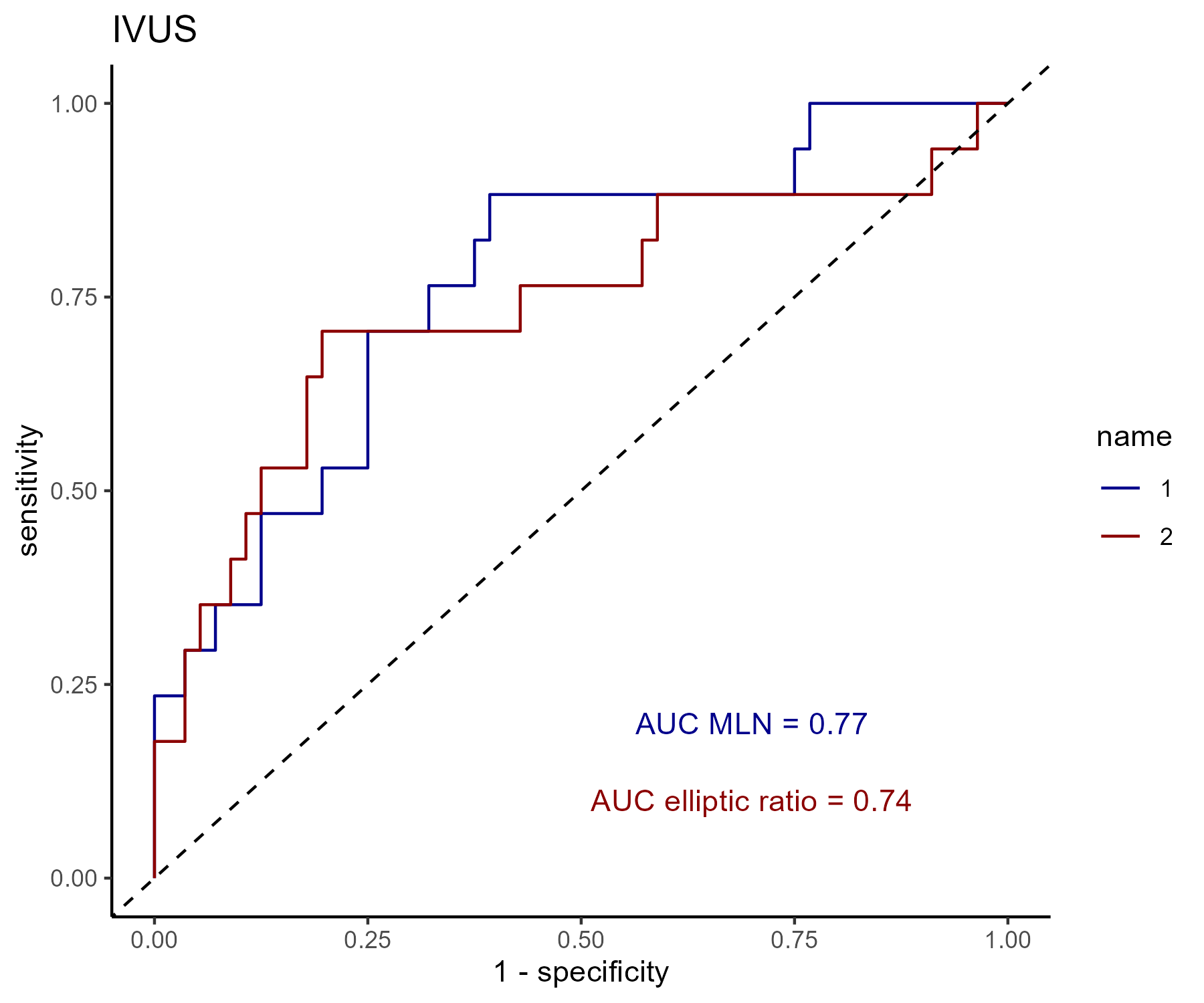


**Supplemental Figure 3:** Relationship of IVUS minimal lumen area during rest and FFR during dobutamine-atropine-volume challenge. IVUS minimal lumen area during rest versus FFR_Dobutamine_. Percent stenosis is indicated by dot size and elliptic ratio by color gradient. The grey line was fitted with a loess function. Abbreviations: FFR_Dobutamine_ = Fractional flow reserve during dobutamine-atropine and volume challenge, IVUS = Intravascular ultrasound during rest, MLN = Maximal lumen narrowing.


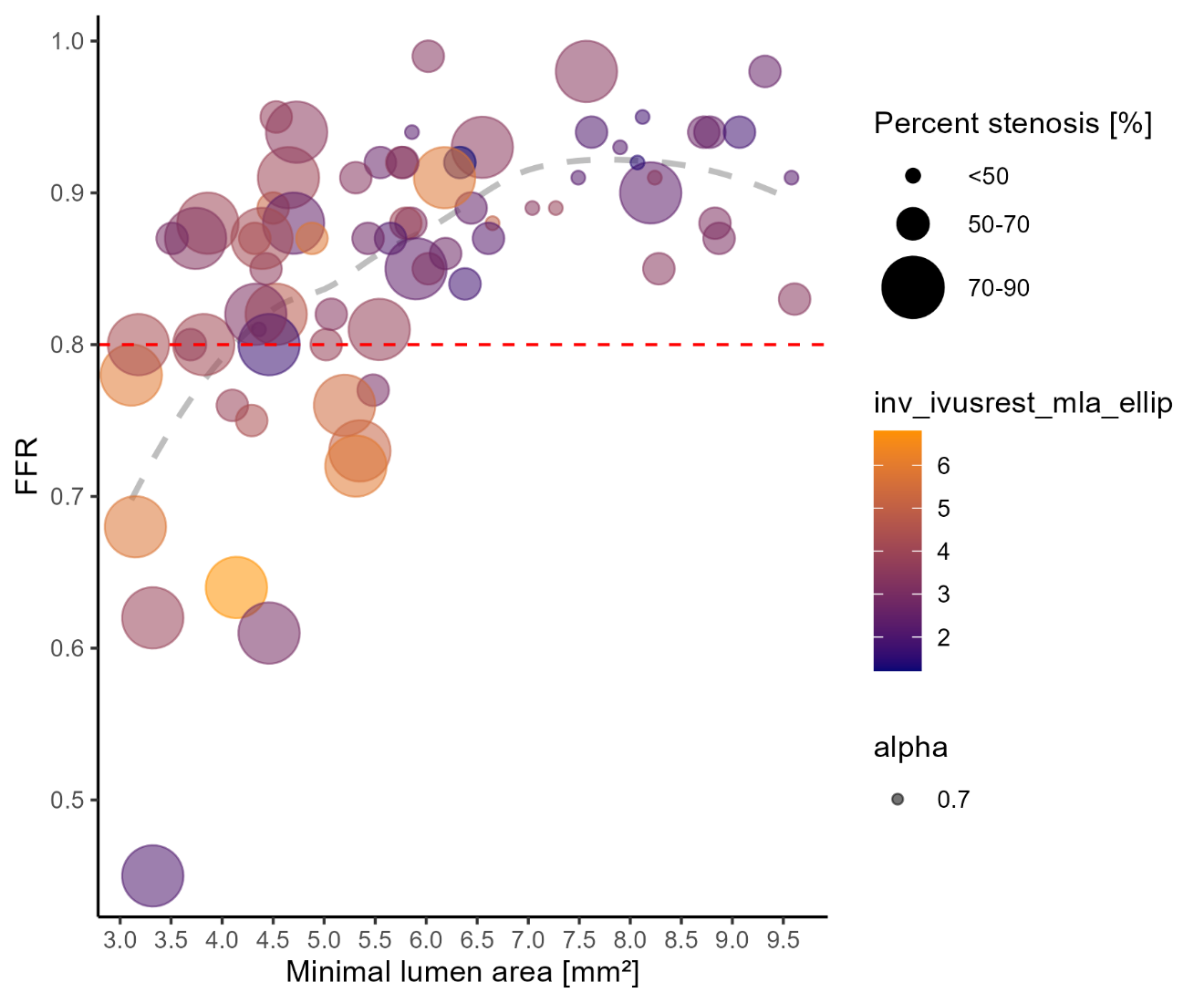


**Supplemental Figure 4:** Relationship of IVUS maximal lumen narrowing during rest and FFR during dobutamine-atropine-volume challenge. Maximal lumen narrowing versus FFR_Dobutamine_. IVUS-MLA categories are indicated by dot size and elliptic ratio by color gradient. The grey line was fitted with a loess function. Abbreviations: FFR_Dobutamine_ = Fractional flow reserve during dobutamine-atropine and volume challenge, IVUS = Intravascular ultrasound during rest, MLA = Minimal lumen area.


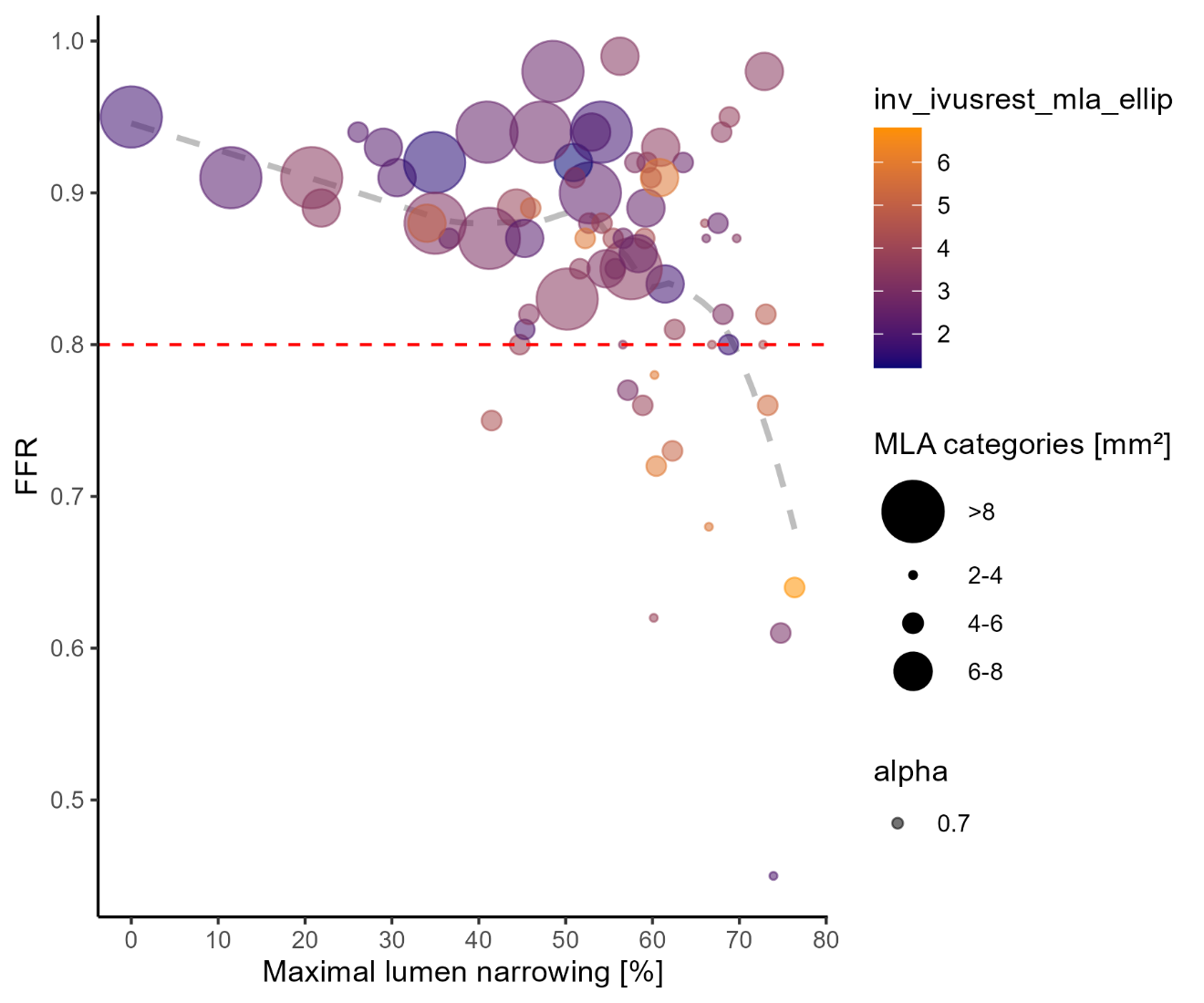


**Supplemental Figure 5:** Relationship of elliptic ratio during rest and FFR during dobutamine-atropine-volume challenge. Elliptic ratio during rest versus FFR_Dobutamine_. IVUS-MLA categories are indicated by dot size and maximal lumen narrowing by color gradient. The grey line was fitted with a loess function. Abbreviations: FFR_Dobutamine_ = Fractional flow reserve during dobutamine-atropine and volume challenge, IVUS = Intravascular ultrasound during rest, MLA = Minimal lumen area, MLN = Maximal lumen narrowing.


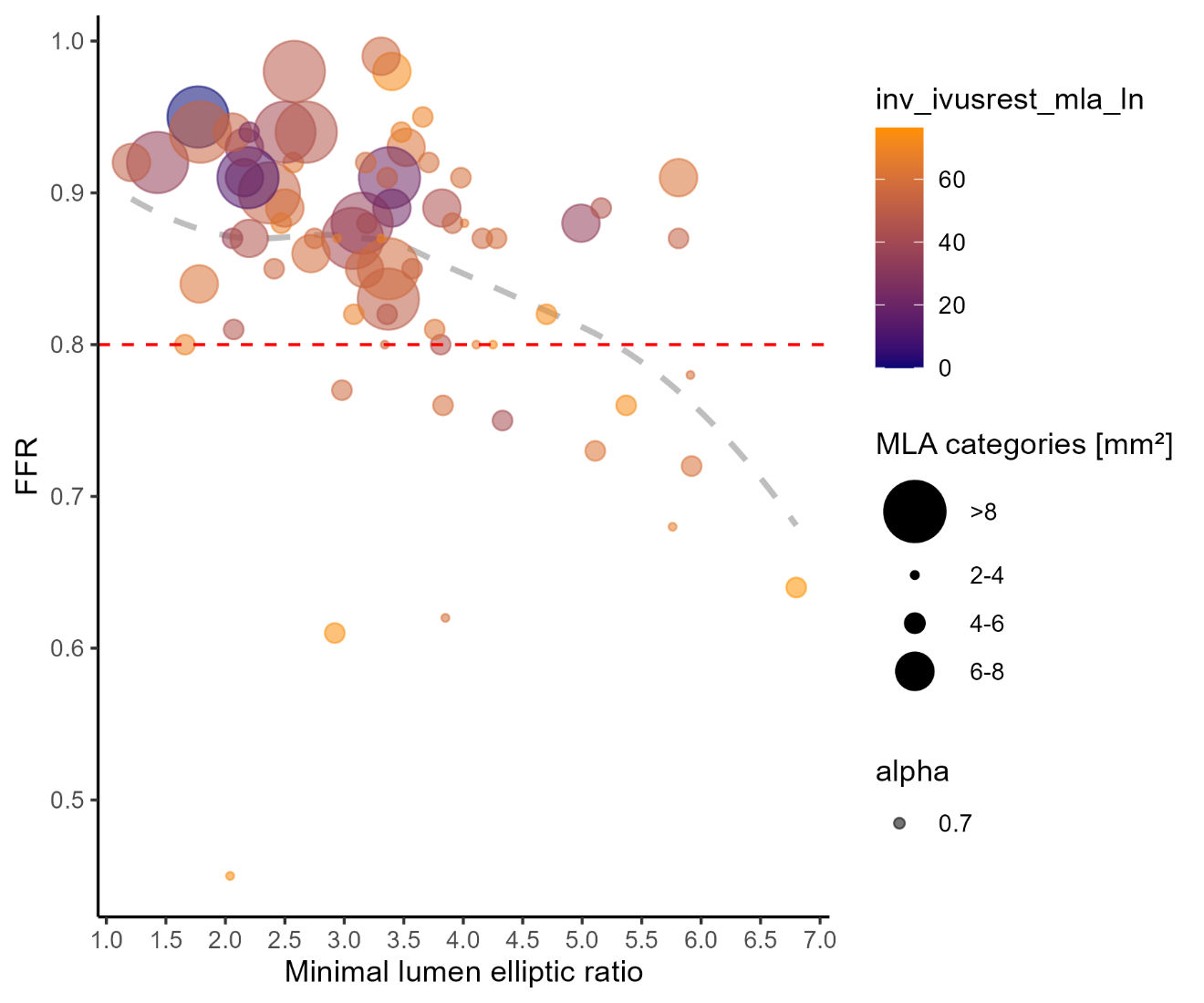


**Supplemental Tables**

**Supplemental Table 1:** Changes from rest to stress (i.e. dobutamine-atropine-volume challenge). Changes from rest condition to dobutamine-atropine-volume challenge.

Abbreviations: FFR = Fractional flow reserve, FFR_Adenosine_ = Fractional flow reserve during adenosine.

|  | **Hemodynamic relevant (FFR_Dobutamine_ ≤0.8) N = 17** | | | **Hemodynamic non-relevant (FFR_Dobutamine_ >0.8) N = 56** | | | Comparison hemo-dynamic relevant vs. non-relevant |
| --- | --- | --- | --- | --- | --- | --- | --- |
| **Variable** | **Rest** | **Dobutamine** | **p-value (adjusted)** | **Rest** | **Dobutamine** | **p-value (adjusted)** | **p-value**  **(adjusted)** |
| Heart rate [bpm] | 74 (67-79) | 150 (147-157) | **<0.001** | 76 (69-89) | 154 (144-161) | **<0.001** | 0.60 |
| N with heartrate >85% maximal heartrate |  | 11/17 (65%) |  |  | 44/56 (79%) |  | 0.40 |
| Systolic aortic pressure [mmHg] | 109 ± 22 | 147 ± 30 | **<0.001** | 113 ± 21 | 143 ± 25 | **<0.001** | 0.28 |
| Diastolic aortic pressure [mmHg] | 67 ± 13 | 75 ± 23 | 0.049 | 68 ± 11 | 71 ± 19 | 0.23 | 0.29 |
| Mean aortic pressure [mmHg] | 81 ± 16 | 99 ± 25 | **<0.001** | 83 ± 12 | 95 ± 20 | **<0.001** | 0.28 |
| FFR | 0.84 (0.80-0.90)* | 0.76 (0.68-0.80) | **0.001** | 0.93±0.04* | 0.89±0.04 | **<0.001** | **0.003** |
| n FFR ≤0.8 | 5 (7 %)* | 17 (23%) | **<0.001** | 0 (0%)* | 0 (0%) | NA | NA |

* FFR_Adenosine_

**Supplemental Table 2:** Averaged results for IVUS and FFR_Adenosine_ as predictors to exclude FFR_Dobutamine_ >0.8 with a 58 patients / 15 patients split for training and test data and 100 random seeds. The table below presents the averaged performance metrics (mean ± SD) from the 100-iteration internal validation described in the Statistical Analysis section of the main manuscript. Three cut-off values were derived per predictor using the Youden’s, maximum sensitivity, and maximum specificity criteria on the training split. The cut-offs were then applied to the corresponding test splits to generate the following results..

| **Predictor** | **Cut-off value** | **AUC** | **Accuracy [%]** | **Sensitivity [%]** | **NPV [%]** | **Specificity [%]** | **PPV [%]** | **n true negative** | **n false negative** | **n false positive** | **n true positive** |
| --- | --- | --- | --- | --- | --- | --- | --- | --- | --- | --- | --- |
| IVUS-MLA [mm^2^] | 8.37 ± 0.58 | 0.91 ± 0.06 | 27 ± 0 | 100 ± 0 | 100 ± 0 | 8 ± 0 | 21 ± 0 | 1 ± 0 | 0 ± 0 | 11 ± 0 | 3 ± 0 |
|  | 4.96 ± 0.56 | 0.9 ± 0.08 | 82 ± 11 | 99 ± 5 | 100 ± 1 | 80 ± 14 | 57 ± 17 | 9 ± 2 | 0 ± 0 | 3 ± 2 | 3 ± 0 |
|  | 3.93 ± 0.48 | 0.9 ± 0.08 | 87 ± 7 | 99 ± 3 | 90 ± 6 | 55 ± 26 | 75 ± 24 | 11 ± 1 | 1 ± 1 | 1 ± 1 | 2 ± 1 |
| Elliptic ratio | 2.7 ± 1.47 | 0.71 ± 0.15 | 38 ± 23 | 94 ± 13 | 89 ± 26 | 7 ± 4 | 28 ± 25 | 3 ± 4 | 1 ± 1 | 9 ± 4 | 2 ± 1 |
|  | 3.72 ± 0.92 | 0.73 ± 0.15 | 70 ± 22 | 80 ± 19 | 89 ± 18 | 81 ± 17 | 48 ± 24 | 8 ± 3 | 1 ± 1 | 4 ± 3 | 2 ± 1 |
|  | 4.72 ± 1.32 | 0.74 ± 0.15 | 75 ± 21 | 96 ± 5 | 83 ± 16 | 40 ± 25 | 57 ± 37 | 10 ± 3 | 2 ± 1 | 2 ± 3 | 1 ± 1 |
| IVUS-MLN [%] | 39.7 ± 14.35 | 0.78 ± 0.16 | 33 ± 17 | 98 ± 8 | 95 ± 15 | 8 ± 2 | 22 ± 14 | 2 ± 3 | 0 ± 1 | 10 ± 3 | 3 ± 1 |
|  | 59.44 ± 6.07 | 0.79 ± 0.15 | 74 ± 16 | 90 ± 16 | 96 ± 8 | 75 ± 17 | 48 ± 19 | 8 ± 2 | 0 ± 1 | 4 ± 2 | 3 ± 1 |
|  | 67.6 ± 8.84 | 0.79 ± 0.14 | 81 ± 16 | 98 ± 4 | 88 ± 7 | 45 ± 25 | 64 ± 29 | 11 ± 3 | 1 ± 1 | 1 ± 3 | 2 ± 1 |
| FFR_Adenosine_ | 0.95 ± 0.04 | 0.78 ± 0.16 | 34 ± 16 | 96 ± 11 | 96 ± 10 | 10 ± 5 | 25 ± 13 | 2 ± 3 | 0 ± 1 | 10 ± 3 | 3 ± 1 |
|  | 0.89 ± 0.04 | 0.82 ± 0.15 | 77 ± 17 | 83 ± 19 | 94 ± 7 | 85 ± 16 | 55 ± 23 | 9 ± 3 | 1 ± 1 | 3 ± 3 | 2 ± 1 |
|  | 0.83 ± 0.09 | 0.83 ± 0.14 | 85 ± 12 | 99 ± 4 | 90 ± 6 | 53 ± 26 | 72 ± 26 | 11 ± 2 | 1 ± 1 | 1 ± 2 | 2 ± 1 |

Abbreviations: AUC = Area under the curve, FFR_Adenosine_ = Fractional flow reserve during adenosine, IVUS = intravascular ultrasound during rest, IVUS-MLA = Minimal lumen area, IVUS-MLN = Maximal lumen narrowing, NPV = Negative predictive value, PPV = Positive predictive value.
